# A Dietary Ketone Ester Normalizes Abnormal Behavior in a Mouse Model of Alzheimer’s Disease

**DOI:** 10.3390/ijms21031044

**Published:** 2020-02-04

**Authors:** Robert J. Pawlosky, Yoshihero Kashiwaya, M. Todd King, Richard L. Veech

**Affiliations:** Laboratory of Metabolic Control, National Institute on Alcohol Abuse and Alcoholism, National Institutes of Health, 5625 Fishers Lane, Bethesda, MD 20892, USA; ykashi.wkmbx1@gmail.com (Y.K.); todd.king@mail.nih.gov (M.T.K.); rveech@mail.nih.gov (R.L.V.)

**Keywords:** ketone bodies, hippocampus, TCA cycle, mitochondria, pyruvate dehydrogenase, anxiety, n-acetyl-aspartate, ketosis, insulin, glucose metabolism

## Abstract

Because of a decreased sensitivity toward insulin, a key regulator of pyruvate dehydrogenase (PDH), Alzheimer’s patients have lower brain glucose utilization with reductions in Tricarboxylic Acid (TCA) cycle metabolites such as citrate, a precursor to n-acetyl-aspartate. In the 3xTgAd mouse model of Alzheimer’s disease (AD), aging mice also demonstrate low brain glucose metabolism. Ketone metabolism can overcome PDH inhibition and restore TCA cycle metabolites, thereby enhancing amino acid biosynthesis. A ketone ester of d-β-hydroxybutyrate was incorporated into a diet (Ket) and fed to 3xTgAd mice. A control group was fed a calorically matched diet (Cho). At 15 months of age, the exploratory and avoidance-related behavior patterns of the mice were evaluated. At 16.5 months of age, the animals were euthanized, and their hippocampi were analyzed for citrate, α-ketoglutarate, and amino acids. In the hippocampi of the Ket-fed mice, there were higher concentrations of citrate and α-ketoglutarate as well as higher concentrations of glutamate, aspartate and n-acetyl-aspartate compared with controls. There were positive associations between (1) concentrations of aspartate and n-acetyl-aspartate (n = 14, R = 0.9327), and (2) α-ketoglutarate and glutamate (n = 14, R = 0.8521) in animals maintained on either diet. Hippocampal n-acetyl-aspartate predicted the outcome of several exploratory and avoidance-related behaviors. Ketosis restored citrate and α-ketoglutarate in the hippocampi of aging mice. Higher concentrations of n-acetyl-aspartate corresponded with greater exploratory activity and reduced avoidance-related behavior.

## 1. Introduction

Many Alzheimer’s patients develop a decrease in brain glucose utilization prior to demonstrating clinical symptoms. One factor that contributes to low brain glucose use is a decrease in brain sensitivity to insulin [1], a key regulator of pyruvate dehydrogenase (PDH) activity [2,3]. Low insulin sensitivity inhibits PDH function, which in turn decreases substrate flow through the Tricarboxylic Acid (TCA) cycle and diminishes metabolite production. 

The TCA cycle metabolite citrate may be utilized for synthesis of cytosolic acetyl-CoA and oxaloacetate, which is a precursor of aspartic acid. The condensation of acetyl-CoA with aspartate produces n-acetyl aspartate, which is a biomarker associated with neuronal viability [4]. The relative abundance of n-acetyl aspartate, as determined by magnetic resonance spectroscopy, correlates with diminished anxiety-related outcomes in patients with neurological disease [5,6], while its presence in reduced concentrations correlates with advancing age [7] and memory deficits in mouse models [8].

In neural tissue, the mitochondrial metabolism of ketones, β-hydroxybutyrate, and acetoacetate is independent of insulin and thus may overcome PDH inhibition to restore TCA cycle intermediates. Ketones are transported across the blood–brain barrier and the mitochondrial membrane via mono-carboxylate transporters [9]. In a model of Alzheimer’s disease (AD), brains of older triple transgenic (3xTgAd) mice had diminished glucose utilization, yet ketone-metabolizing enzymes increased [10].

In the non-fasted state, blood ketone concentrations are generally below 1 mM. Blood ketone concentration levels become elevated during prolonged fasts through a hormonally-activated cleavage of adipose tissue triglycerides with accompanying release of free fatty acids into the blood, followed by partial oxidation in the liver to β-hydroxybutyrate and acetoacetate. In this study, blood ketone levels in male 3xTgAd mice were elevated using a synthetic dietary ketone ester (Ket) (an ester of d-β-hydroxybutyrate and R-1,3 butane diol). During digestion and absorption, the Ket is hydrolyzed and the 1,3-butane diol undergoes partial oxidation in the liver, forming an additional mole of d-β -hydroxybutyrate. When mice were 8.5 months old, they were divided into two groups; one group was placed on a Ket diet where 21% of their carbohydrate calories were replaced with the ketone ester. Control animals were maintained on an iso-calorically identical diet. At 15 months, exploratory and avoidance-behavior patterns were assessed using an open field trial and an elevated open- and closed-arm plus maze, respectively. At 16.5 months, mice were euthanized, their hippocampi extracted, and citrate, α-ketoglutarate, and amino acids were quantified using stable isotope dilution mass spectrometry [11]. In order to evaluate potential associations between the TCA cycle metabolites and the amino acids, the concentrations of (1) citrate were compared to those of aspartate, (2) aspartate to those of n-acetyl aspartate, and (3) α-ketoglutarate to those of glutamate. In addition, since low levels of hippocampal n-acetyl-aspartate are associated with cognitive impairment and anxiety-related disorders in humans [5,6], potential associations between its concentration and avoidance-related behavior, as evaluated using an elevated plus maze, and exploratory behavior, evaluated in open field tests, were assessed.

## 2. Results

Previously, the long-term effects of feeding the Ket diet on several glycolytic intermediates, TCA cycle metabolites and the cytosolic and mitochondrial redox potentials were described in these 3xTgAd mice [12]. We now extend this analysis to an examination of potential associations between (1) the TCA cycle metabolites citrate and α-ketoglutarate, and (2) the amino acids aspartate, n-acetyl aspartate and glutamate. Further, for mice at 15 months of age, we investigated potential associations between these neural chemicals and (1) exploratory behavior (distance traveled, ambulatory time, and ambulatory counts) using an open field area, and (2) avoidance-related behavior using an elevated open- and closed-arm plus maze.

**Figure 1:**
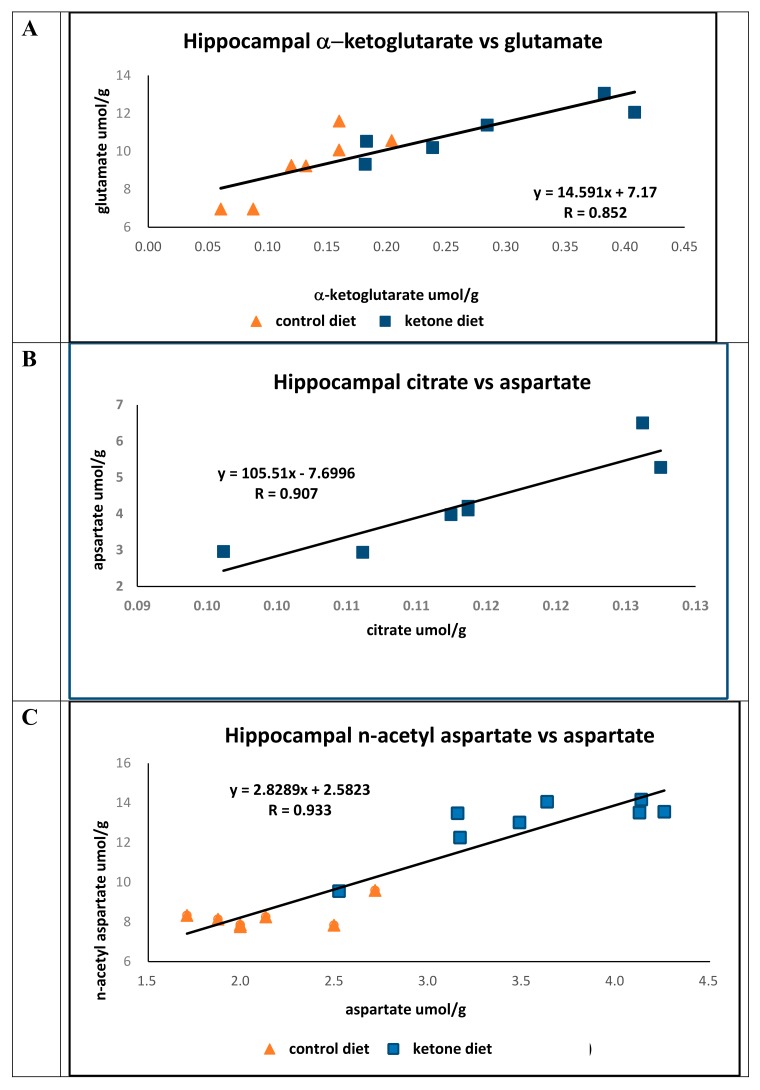
Pearson’s linear correlation comparing concentrations of hippocampal TCA cycle metabolites with their respective amino acids from 3xTgAd mice fed either a control diet (n = 7, triangle) or a ketone ester diet (*n* = 7, square) for 8 months, measured at 16.5 months of age. (**A**) Concentrations of α-ketoglutarate versus glutamate, (**B**) concentrations of citrate versus aspartate, and (**C**) concentrations of aspartate versus n-acetyl aspartate.

Blood ketone levels were significantly elevated in mice fed the Ket diet (Cho, 0.14 mM ± 0.01; Ket, 0.72 ± 0.13 mM; *p* < 0.01), and the concentration of hippocampal α-ketoglutarate was greater in mice fed the Ket diet (Cho, 0.147 ± 0.039 μmol/g; Ket, 0.280 ± 0.089 μmol/g; *p* < 0.006). However, glutamate concentrations were only marginally elevated in Ket-fed mice compared to mice on the Cho diet (Cho, 9.6 ± 1.16 μmol/g; Ket, 11.1 ± 1.23 μmol/g; *p* < 0.05). In both groups, there were strong positive correlations (R = 0.852) between hippocampal α-ketoglutarate and glutamic acid (Figure 1A).

The concentrations of citrate (Cho, 0.132 ± 0.0128 μmol/g; Ket, 0.264 ± 0.098, μmol/g; *p* < 0.04) and aspartate (Cho, 2.13 ± 0.35 μmol/g; Ket, 4.11 ± 0.51 μmol/g; *p* < 0.001) were significantly greater in mice fed the Ket diet compared to mice on the control diet. While there was a strong association between the concentrations of citrate to that of aspartate in mice on the Ket diet (N = 7, R = 0.907) (Figure 1B) no association was found between citrate and aspartate in the control group (N = 7, R = 0.107). The lack of correlation between citrate and aspartate in the control group is not immediately clear; however, it does suggest that there may be fewer healthy and/or viable neurons in this brain region.

The hippocampal concentration of n-acetyl-aspartate in mice fed the Ket diet was significantly greater than that of the controls (Cho, 8.42 ± 0.65 μmol/g; Ket, 13.0 ± 1.26 μmol/g; *p* < 0.0001), and there was a strong positive association between aspartate and *n*-acetyl-aspartate observed across both dietary groups (*R* = 0.933) (Figure 1C).

**Figure 2:**
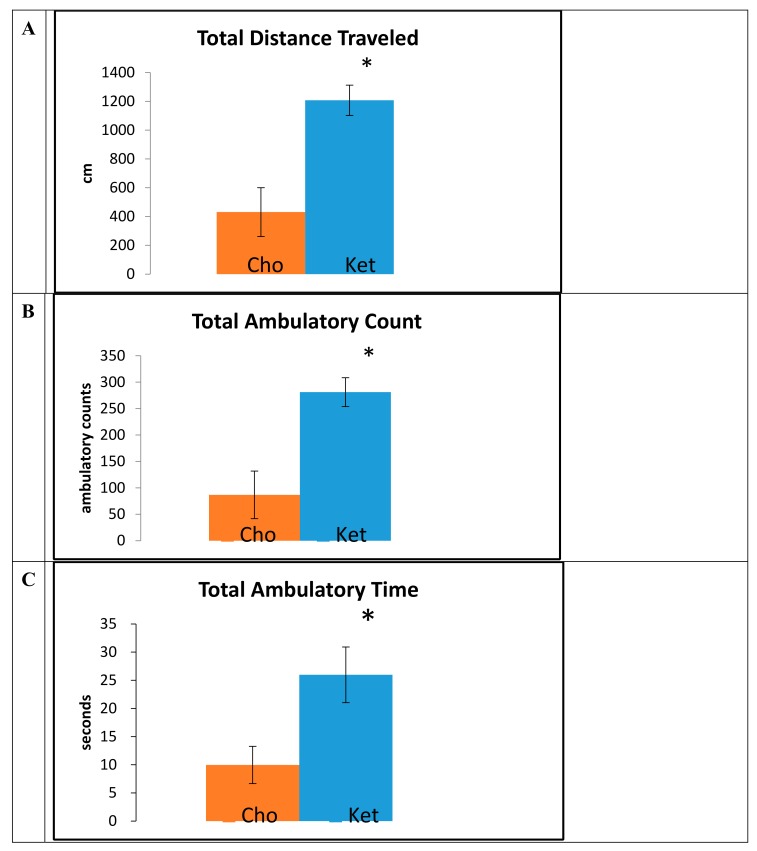
Open field assessments for 3xTgAd mice fed either a control diet (n = 7, Cho) or ketone ester diet (n = 7 or 8, Ket) for 8 months, measured at 15 months of age. (**A**) Total distance traveled, (**B**) total ambulatory counts, and (**C**) total ambulatory time.

Data obtained from the open field trials, measured at 15 months of age, demonstrated that mice maintained on the Ket diet traveled greater total distances (Cho, 414 ± 233 cm; Ket, 1140 ± 164 cm; *p* < 0.001) (Figure 2A), had a greater number of ambulatory counts (Cho, 89 ± 44; Ket, 265 ± 35; *p* < 0.001) (Figure 2B), and had increased total ambulatory time (Cho, 9.6 ± 3.2 sec; Ket, 27.4 ± 6.8 sec; *p* < 0.001) (Figure 2C) compared to controls.

**Figure 3:**
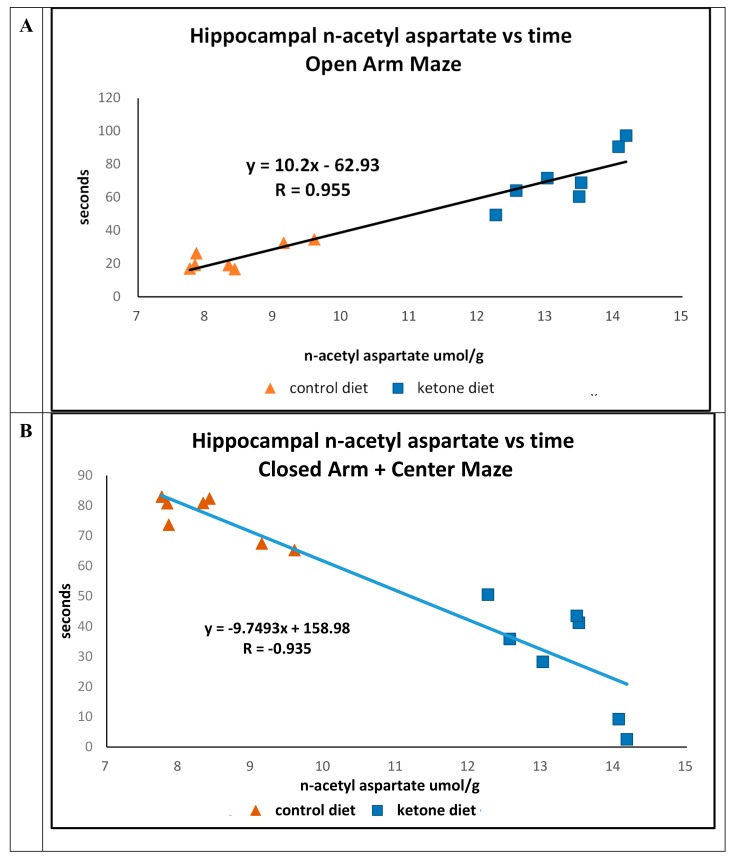
Elevated open- and closed-arm maze assessments of 3xTgAd mice fed either a control diet (n = 7, triangle) or ketone ester diet (n = 7, square) for 8 months, measured at 15 months of age. (**A**) Linear correlations comparing concentrations of hippocampal n-acetyl aspartate (assayed at 16.5 months) versus time spent in the open-arm portion of the maze, in seconds. (**B**) Hippocampal n-acetyl aspartate versus time spent in the closed-arm and center portions of the maze.

Previously, we reported that mice maintained on the Ket diet were less likely to occupy the closed-arm and center portions (Ket, 47.3 s. Cho, 74.2 s.; *p* = 0.01) of the elevated platform plus maze compared with time spent in the open-arm portions (Ket, 52.2 s.; Cho, 26.0 s.; *p* = 0.011) [13]. We now report that there were strong positive (R = 0.955) (time in open arm) and negative (R = -0.935) (closed arm) associations between the concentrations of n-acetyl in the hippocampus versus time in animals maintained on either of the diets (Figure 3A,B).

**Figure 4:**
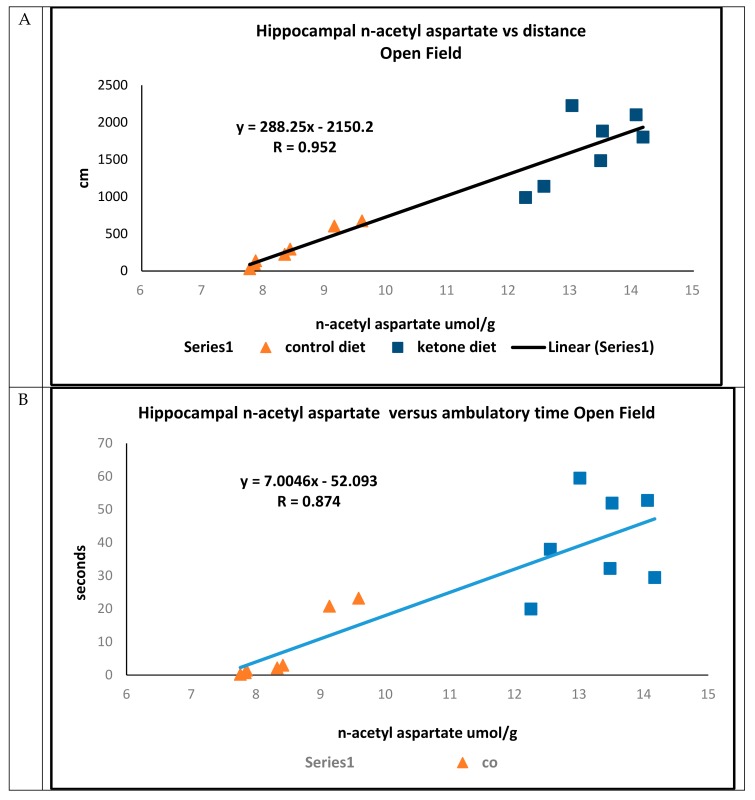

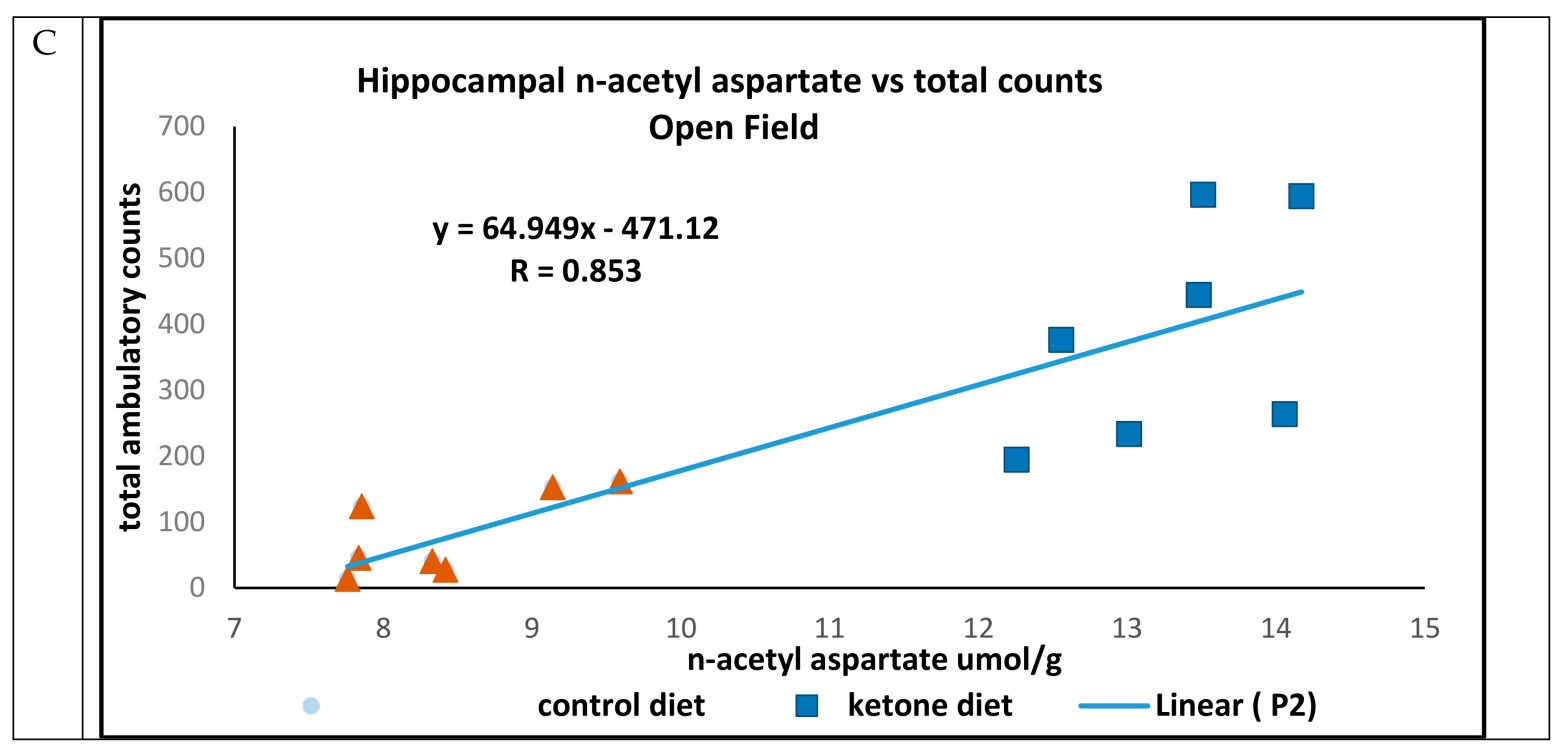
Open field assessments of 3xTgAd mice fed either a control diet (n = 7, triangle) or ketone ester diet (n = 7, square) for 8 months, measured at 15 months of age. (**A**) Linear correlations comparing concentrations of hippocampal n-acetyl aspartate (assayed at 16.5 months of age) versus total distance traveled, (**B**) hippocampal n-acetyl aspartate versus ambulatory time, and (**C**) hippocampal n-acetyl aspartate versus ambulatory counts.

In open field trials, there were strong positive associations between concentrations of hippocampal n-acetyl aspartate and total distance traveled (R = 0.952) (Figure 4A), ambulatory time (R = 0.874) (Figure 4B), and ambulatory counts (R = 0.853) (Figure 4C) in mice maintained on either the Cho or Ket diets.

## 3. Discussion

PET studies using a radio-labeled glucose analog (e.g., Fluoro-deoxyglucose (Fdg)) have consistently demonstrated a low rate of glucose metabolism (~20%–30% lower) in brain regions involved in memory processing (e.g., the hippocampus, posterior cingulate, temporal, and parietal lobes) in Alzheimer’s patients [14,15]. Decreased cerebral blood flow, insulin resistance, inhibition of GLUT (Glucose) transporters and glycolytic enzymes, and amyloid-stimulated PDH phosphorylation all contribute to decreased glucose metabolism in clinical AD and transgenic models [14,15,16,17]. In the 3xTgAd transgenic mouse model, Ding et al. observed a lower uptake of fDG and an elevated level of the neuronal monocarboxylate transporter, MCT 2, in the aged hippocampus [18]. Also, Yao et al. observed an increase in the expression of the ketone-metabolizing enzymes succinyl-CoA:3-ketoacid CoA transferase (SCOT) and acetyl-CoA acetyltransferase 1 (ACAT1) in the same transgenic species maintained on a diet intended to induce mild ketosis [19].

To our knowledge, this is the first report in a model of a major neurological disease that demonstrates that a therapeutic treatment can correct low brain glucose metabolism and furnish the mitochondria with TCA cycle substrates, and also elevate the concentration of a biomarker correlated with improved behavioral outcomes. In the hippocampal region of ketotic mice, there were significantly higher concentrations of citrate and α-ketoglutarate, precursors to the amino acids aspartate and glutamate, respectively. Meanwhile, the concentration of hippocampal α-ketoglutarate was strongly correlated to that of glutamate in mice on either diet, and only in mice fed the Ket diet was there an association between the concentration of citrate and that of aspartate. Concentrations of hippocampal n-acetyl-aspartate, a biomarker of neuronal viability, were greater in mice fed the Ket diet, and the concentration of this amino acid was firmly linked to that of aspartate in both Cho- and Ket-fed mice.

N-acetyl-aspartate is a biomarker in the diagnosis of cognitive function in Alzheimer’s patients [6] and anxiety-related disorders in Parkinson’s patients [5], while reduced concentrations are associated with aging [7] and memory deficits [8] in animal models. The higher concentrations of n-acetyl aspartate observed in the present study follow from the increased availability of the TCA cycle-derived acetyl-coA and carbon units from aspartate via oxaloacetate in Ket-fed mice. We demonstrated that mildly ketotic mice had a much greater degree of exploratory behavior, as assessed by total distance traveled, ambulatory time, and ambulatory counts in an open field trial at 15 months of age, compared to mice on the control diet. We also demonstrated that mice on the Ket diet were less anxious and more likely to remain in the open arms of the elevated plus maze than in the closed regions, and these findings were also associated with the concentration of hippocampal n-acetyl aspartate. There were no strong linkages between other neurochemicals assayed and these behavioral outcomes (data not shown).

Results from this study strongly suggest that mild ketosis should have positive benefits for the treatment of Alzheimer’s patients, by supplying substrates to the TCA cycle and correcting metabolic deficiencies due to insulin insensitivity, PDH inhibition, or low glucose metabolism. The results further suggest that the concentration of an important biomarker of anxiety and cognitive impairment in the hippocampus, n-acetyl-aspartate [5,6], is closely associated with TCA cycle metabolism and its concentration may be enhanced using therapeutic ketosis.

## 4. Materials and Methods

### 4.1. Animal Procedures, Diets, and Tissue Harvesting

All experiments were reviewed and approved by the Animal Care and Use Committee of NIAAA (National Institute on Alcohol Abuse and Alcoholism), NIH (National Institutes of Health), Project ID code NIA-018-2011, approved on 10 October 2011. As reported previously, there were 11–15 3xTgAD male mice housed in groups of 2–3 animals per cage [13]. At 8.5 months of age, mice were randomly assigned to one of the 2 dietary groups (Ket or Cho). The Cho and Ket diets have been fully described previously [13]. Briefly, the Cho diet was based on the American Institute of Nutrition-1993 (AIN-93) diet for laboratory rodents. The sole difference between the Cho diet and the Ket diet was the amount of corn starch in either of the two diets and the addition of the ketone ester in the Ket diet. The control diet contained 137 g corn starch/1000 g of diet and 0 g Ket. The Ket diet contained 85 g starch/1000 g of diet and 125 g Ket/1000 g of diet. The Ket was synthesized from d-β-hydroxybutyrate and R-1,3-butanediol [13]. Animals were fed ad libitum on either diet, beginning at 8.5 months of age for an additional 8 months.

### 4.2. Open Field Testing

Spontaneous activity of mice in an open field test was quantified using the MEDOFA-MS system (Med Associates, St. Albans, VT, USA). The motion of a mouse was traced with infrared light-sensitive photocells with the apparatus placed in a 120-lx ventilated box. The dimensions of the arena were 40.6 cm × 40.6 cm; the inner 20.32 cm was defined as the center zone, and outside this was defined as the peripheral zone. Mice were placed in the center of the open field and assessed by measuring ambulatory counts, ambulatory time, and total distance covered over a period of 10 min.

### 4.3. Elevated Plus Maze Testing

The apparatus consisted of a plus sign-shaped maze, elevated 60 cm from the floor, with each arm measuring 25 cm × 5 cm. Two arms lacked side walls (open arms), and two arms were enclosed in 30-cm high walls (closed arms). Each mouse was placed in the middle of the maze facing an open arm. After 5 min of testing, mice were returned to their home cages. Arm preference was automatically analyzed using the ANY-maze video tracking software (Stoelting, Wood Dale, IL, USA), and time spent in each arm was recorded. Elevated plus maze experiments were performed under a light intensity of 1300 lx.

Mice were euthanized at 16.5 months of age, and their hippocampi were removed, immediately frozen in liquid nitrogen, and stored at −80 °C until analysis. The frozen brain tissue was then used for metabolite assays as described below.

### 4.4. Materials, Reagents and Instrumentation

Stable isotopically-labeled (^13^C- and ^2^H-) organic acid standards (citrate, α-ketoglutarate) and amino acids were procured from CDN Isotopes (CDN Isotopes, Pointe-Claire, Quebec, Canada). The *N*-methyl-*N*-(tert-butylmethylsilyl) trifluoroacetamide (MTBSTFA) with 1% tert-butyldimethylchlorosilane (TBDMCS) reagent and the bis-trimethylfluoro methyl silyl (BSTFA) were purchased from Pierce (Pierce Chemical Co., Rockford, IL, USA). Samples were analyzed on an Agilent 5973 quadrupole GC-MS (Agilent, Wilmington, DE, USA) [12].

### 4.5. Blood Ketone Analysis

Thawed mouse plasma was analyzed for β-hydroxybutyrate using glucose and ketone sticks and a Precision-Xtra meter (Abbott Labs, Abbott Park, IL, USA) according to previously described methods [13].

### 4.6. Perchloric Acid (PCA) Extraction

Frozen brain tissue (20–40 mg) was extracted into a 3.6% PCA solution and neutralized with KHCO_3_ (3 M) according to previously described methods [12]. The final volume of the extract was 100 μL.

### 4.7. Gas Chromatography–Mass Spectrometry of Organic and Amino Acids

Twenty microliters of the brain tissue extract was taken for determination of citrate, α-ketoglutarate, glutamate, aspartate, and n-acetyl aspartate, with a commensurate amount of the C_13_- or ^2^H- labeled internal standards (2 to 3-fold in excess of the endogenous metabolite) added, for quantitative analysis by gas chromatography–mass spectrometry [12]. The aqueous solution of the sample specimens was reduced to dryness under a stream of nitrogen (99.99%) and reacted with tertiary butyl dimethyl silyl chloride to form silyl esters and ethers according to previously described methods [12]. One microliter of the derivatized sample was injected onto a 30-meter capillary gas chromatograph column and analyzed in the electron impact mode on a quadrupole mass spectrometer. Individual metabolites were quantified using the ratio of the area counts of the most prominent ion fragments of the unlabeled compound referenced to the analogous ion of the labeled internal standards.

### 4.8. Statistics

All data values are given as mean ± SEM. Statistical differences were determined by Student’s t-test and Pearson’s linear correlation in GraphPad Prism 6. In all cases, statistical significance was set at *p* ≤ 0.05.

## Abbreviations

PDH:	pyruvate dehydrogenase
Ket:	ketone ester—an ester of D-β-hydroxybutyrate and R-1,3 butane diol
Cho:	control diet
